# The effects of weather factors and altitude on physical and technical performance in professional soccer: A systematic review

**DOI:** 10.1016/j.jsampl.2022.100002

**Published:** 2022-08-21

**Authors:** Sarah Illmer, Frank Daumann

**Affiliations:** Chair of Sports Economics and Health Economics, Institute for Sports Science, Faculty of Social and Behavioural Sciences, Friedrich Schiller University Jena, Jena, Germany

**Keywords:** Weather, Temperature, Humidity, Altitude, Performance, Adaptation

## Abstract

Soccer, as the most popular sport in the world, is characterized by complex performance requirements and is influenced by many external factors. In order to record and systematize the scientific findings of the effects of weather factors and altitude on physical and technical performance in professional male and female soccer a systematic literature search was conducted in the relevant databases from 8th to 15th of February 2022. From 2.396 records, 150 were selected for detailed screening. 21 studies were included in this review that met the following inclusion criteria: professional male or female soccer players over 18 years of age; field study under real-life conditions; effects on physical and/or technical performance, influence of at least one weather-related factor. The selected articles considered different research objects, periods of time, technologies, or methods. Most publications investigated the factors of temperature, humidity and altitude and showed some significant effects on physical performance, while technical performance often did not change significantly. For all analysed environmental factors, it can be summarized that in different environmental conditions, professional soccer players may consciously adjust certain performance parameters to maintain key match characteristics throughout the whole game. This pacing strategy allows them to keep the influence of environmental factors in check as far as possible.

## 1 Introduction

As the world's most popular team sport, soccer is played in every country across the world [7]. Soccer is a very complex and high-intensity sport in which players perform varying activities over 90 ​min, characterized primarily by physical performance like total distance, high-intensity running or number of sprints and technical performance like passes, ball possession or goals scored per game [33]. FIFA, as the highest institution in international soccer, postulates that increased running performance is associated with a more offensive style of play and consequently increases the attractiveness of the game, which has already been empirically proven [36]. In addition to the complexity of sporting performance, there are other factors that influence success in international professional soccer. One of them is weather as an important indicator of football success at the international level [17,18,21,32].

When professional soccer players compete at an international level, they are exposed to different weather conditions to which they must adapt in the best possible way to achieve optimal performance [7]. While less extreme weather conditions prevail in Europe, professional soccer players are faced with higher challenges especially during the World Cups, held every four years on different continents. For example, the 2014 FIFA World Cup in Brazil was criticized for exposing players to excessive heat [25] and the upcoming 2022 World Cup, to be held in Qatar, is also expected to experience extreme temperatures that can have a decisive impact on player performance even if it is moved to the winter [33].

Numerous laboratory studies (RCT) have already been conducted to investigate the physical performance of an athlete under different weather conditions. These simulated studies mostly relate to athletes in general, but some also to specific sports like soccer. However, fewer real-world field studies investigating weather effects in experimental match-play or in real competitions have been conducted to date. For this reason, studies on physical performance in soccer also dominate and there are significantly fewer results on effects on technical performance. The effects on both physical and technical performance are considered in this review. The publications on weather effects in soccer provide interesting and partly controversial results, which, however, have not been sufficiently systematized and compared so far. The aim of this paper is to identify and evaluate these studies through a systematic database search and to discuss the results. The following central research questions should be answered at the end of this systematic review: How does the physical and technical performance of professional soccer players change under the influence of selected environmental factors? Furthermore, with the help of this review, existing research gaps in this topic will be discovered in order to build further research on them.

## 2 Methods

**Fig. 1:**
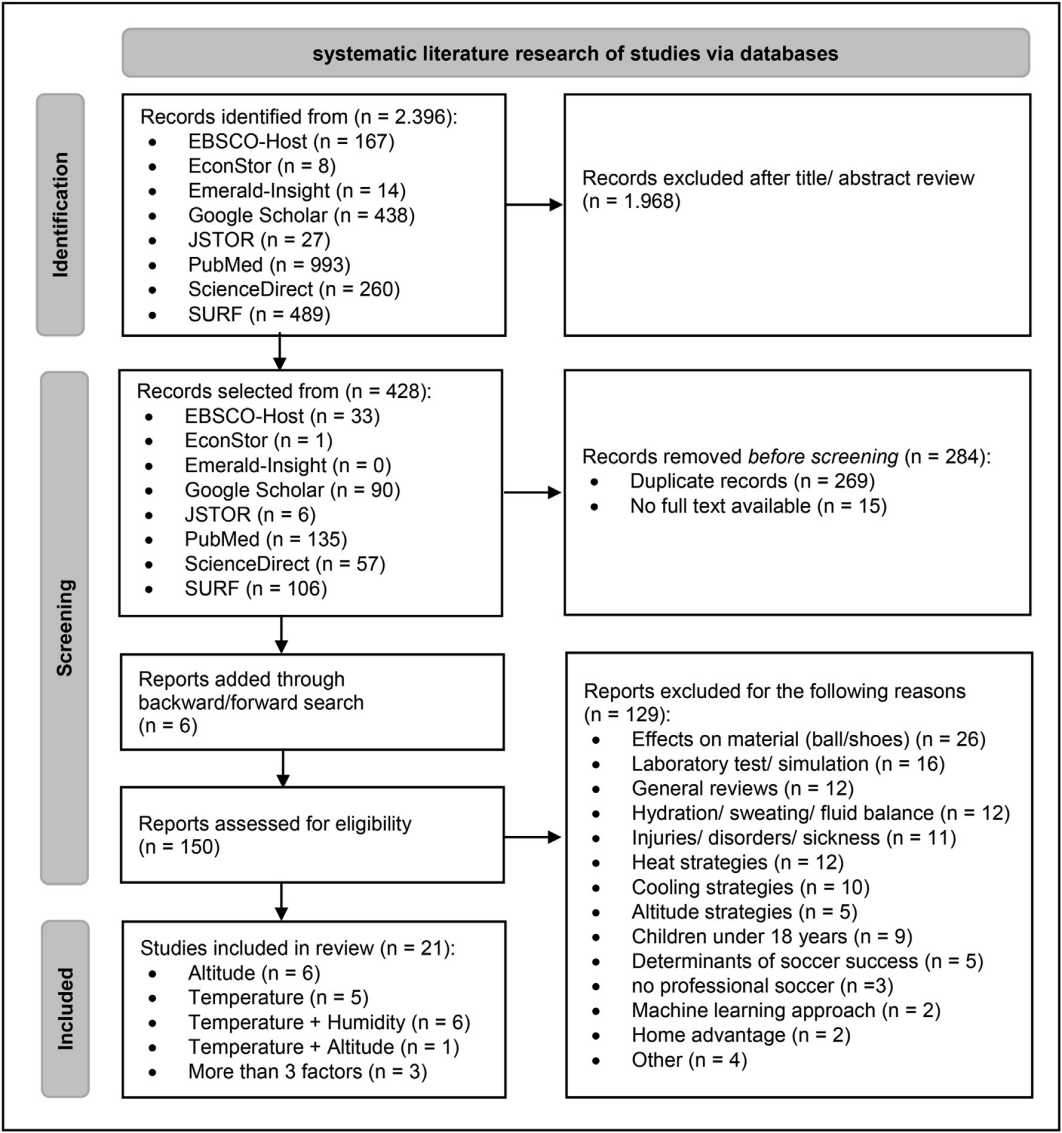
PRISMA flow diagram for study selection in systematic reviews.

This systematic review examines the effects of selected weather-related factors on physical and technical performance in professional soccer. For this purpose, a database search was used to identify relevant studies that met the inclusion criteria. This process is illustrated with the help of a PRISMA diagram (Fig. 1) and explained below. We did not fully adhere to the PRISMA guidelines, as they would have restricted the acquisition of knowledge too much in some places. This is mainly due to the different approaches of the identified studies. For example, the individual studies measured different parameters in terms of both technical and physical performance, so that a synthesis of the studies was not meaningful here.

### 2.1 Database search

In order to cover the current state of research as complete as possible, a search was performed in the databases *EBSCO-Host, EconStor, Emerald-Insight, Google Scholar, JSTOR, PubMed, ScienceDirekt,* and *SURF* from 8th to 15th of February 2022 using the keywords *soccer* and *football* in combination with the terms *weather, temperature, heat, cold, altitude, humidity, wind, sun, air pressure, rain* and *precipitation.* The results and relevant results for every combination of keywords in every database are shown in Table 1, Table 2.

**Table 1:** Database Search with the term *soccer*.

Databases	Soccer and weather		Soccer and temperature		Soccer and heat		Soccer and cold		Soccer and altitude		Soccer and humidity		Soccer and wind		Soccer and sun		Soccer and air pressure		Soccer and rain		Soccer and precipitation		Total	
	Results	Relevant	Results	Relevant	Results	Relevant	Results	Relevant	Results	Relevant	Results	Relevant	Results	Relevant	Results	Relevant	Results	Relevant	Results	Relevant	Results	Relevant	Results	Relevant
EBSCO-Host	12	1	15	4	7	2	8	2	3	1	1	1	4	0	5	3	0	0	0	0	1	1	56	15
EconStor	1	0	0	0	0	0	0	0	1	1	0	0	0	0	0	0	0	0	1	0	1	0	4	1
Emerald-Insight	1	0	1	0	0	0	0	0	1	0	0	0	0	0	0	0	0	0	0	0	1	0	4	0
Google Scholar (without citation, search in title)	6	6	29	9	43	20	32	1	23	12	5	4	7	4	7	1	0	0	1	0	0	0	153	57
JSTOR	2	1	1	0	0	0	1	0	1	1	0	0	0	0	2	1	1	0	1	0	2	1	11	4
PubMed	34	8	136	16	87	19	55	3	39	12	36	16	7	0	23	6	1	0	2	0	2	0	422	71
ScienceDirect	7	3	38	9	16	7	10	1	3	0	9	5	19	12	1	0	5	1	2	0	0	0	110	38
SURF	16	4	54	16	38	11	39	3	20	9	11	5	19	13	3	1	4	0	2	0	0	0	206	62
**Total**	**79**	**23**	**153**	**43**	**119**	**50**	**102**	**9**	**91**	**36**	**62**	**31**	**56**	**29**	**41**	**12**	**11**	**1**	**9**	**0**	**7**	**2**	**730**	**236**

**Table 2:** Database Search with the term *football*.

	Football and weather		Football and temperature		Football and heat		Football and cold		Football and altitude		Football and humid		Football and wind		Football and sun		Football and air pressure		Football and rain		Football and preciptation		Total	
	Results	Relevant	Results	Relevant	Results	Relevant	Results	Relevant	Results	Relevant	Results	Relevant	Results	Relevant	Results	Relevant	Results	Relevant	Results	Relevant	Results	Relevant	Results	Relevant
EBSCO-Host	30	3	21	5	14	4	18	0	5	3	2	2	5	1	8	0	0	0	6	0	2	0	111	18
EconStor	4	0	0	0	0	0	0	0	0	0	0	0	0	0	0	0	0	0	0	0	0	0	4	0
Emerald-Insight	4	0	1	0	0	0	0	0	0	0	1	0	3	0	0	0	0	0	0	0	1	0	10	0
Google Scholar (without citation, search in title)	16	3	41	5	122	12	41	2	29	9	6	2	18	0	7	0	2	0	3	0	0	0	285	33
JSTOR	7	1	0	0	3	0	3	0	1	1	0	0	1	0	1	0	0	0	0	0	0	0	16	2
PubMed	6	3	16	2	66	8	8	2	38	14	45	10	11	1	9	4	1	0	5	1	2	0	207	45
ScienceDirect	15	1	41	4	28	3	15	0	5	0	6	1	29	10	3	0	4	0	4	0	0	0	150	19
SURF	15	2	74	10	79	11	41	4	16	3	20	6	21	7	6	1	4	0	6	0	1	0	283	44
**Total**	**97**	**13**	**194**	**26**	**312**	**38**	**126**	**8**	**94**	**30**	**80**	**21**	**88**	**19**	**34**	**5**	**11**	**0**	**24**	**1**	**6**	**0**	**1066**	**161**

The search was limited to English keywords because the relevant literature on this topic is international. Therefore, only articles written in, or already translated into, English, were considered for further analyses. Also, the articles must be published in a peer-reviewed journal. Further the search was limited to the abstract because the most important information for the selection of relevant studies can be found in the abstract. At this point it should be noted that for Google Scholar there is only the option to limit the search to the title.

From altogether 2.396 results, that showed up in the databases, 1.968 were not considered for screening after title and abstract review, because of no relevant content. From the remaining 428 records 269 were removed because of duplicates and another 15 were removed because there was no full text available. Through backward and forward search in the literature of the remaining studies a further six relevant papers were identified. Finally, 150 reports were assessed for eligibility in the next step.

### 2.2 Study selection

After the database search, the remaining 150 results were re-examined. The entire search process is shown in a PRISMA flow diagram in Fig. 1. Various reasons, that can be taken from the PRISMA diagram, led to the exclusion of a further 129 studies. The most important reasons were: effects on material (ball/shoes) (n ​= ​26), laboratory test/simulation (n ​= ​16), general reviews (n ​= ​12) and studies about the topics hydration/sweating/fluid balance (n ​= ​12).

Finally, 21 studies were included in this review that met the following inclusion criteria. The soccer players must be professional male or female athletes over 18 years of age. Because laboratory studies or simulations were excluded, only field studies under real-life conditions (on the soccer pitch) were included in the review. They must investigate effects on physical and/or technical performance and include the influence of at least one environmental factor e.g. temperature, humidity or altitude.

### 2.3 Quality rating

To assess the quality of the individual papers, nine “yes or no” questions were then answered for each study. The scale used for this purpose was adapted from other systematic reviews [13,35] related to soccer and slightly modified for this topic (Table 3). The 21 papers reviewed scored between five and nine points, which is shown in Table 4. All articles met criterion 1 (peer-reviewed), 2 (indexed journal), 3 (objective(s) is/are clearly set out), 8 (other contextual or situational variables are considered) and 9 (results are clearly presented). The resulting total of 5 points was awarded to those four studies that were the only ones to look exclusively at effects on technical factors and not on physical performance [8,11,26,29]. The other 16 studies considered the number/distribution of recordings/players and 9 studies the duration of player recordings as well. Only in 6 publications a distinction according to the players position was made and the reliability/validity of the instrument is mentioned or is measured in only 5 publications. There is only one study that meets all the criteria and can therefore be attributed the highest quality [34].

**Table 3:** Quality criteria used to analyze the publications.

Q1	The study is published in a peer-reviewed journal or book	No ​= ​0	Yes ​= ​1
**Q2**	The study is published in an indexed journal	No ​= ​0	Yes ​= ​1
**Q3**	The study objective(s) is/are clearly set out	No ​= ​0	Yes ​= ​1
**Q4**	Either the number of recordings is specified or the distribution of players/recordings used is known	No ​= ​0	Yes ​= ​1
**Q5**	The duration of player recordings is clearly indicated	No ​= ​0	Yes ​= ​1
**Q6**	A distinction is made according to player positions	No ​= ​0	Yes ​= ​1
**Q7**	The reliability/validity of the instrument is mentioned or is measured	No ​= ​0	Yes ​= ​1
**Q8**	Other contextual or situational variables (e.g. match status, match location, type of competition or quality of the opponent) are taken into account	No ​= ​0	Yes ​= ​1
**Q9**	The results are clearly presented	No ​= ​0	Yes ​= ​1

**Table 4:** Quality rating of each publication based on the nine quality criteria.

Author (Year)	Q1	Q2	Q3	Q4	Q5	Q6	Q7	Q8	Q9	Total
Aughey et al. (2013)	1	1	1	1	0	0	0	1	1	6
Benjamin et al. [4]	1	1	1	1	1	0	0	1	1	7
Bohner et al. [6]	1	1	1	1	1	0	1	1	1	8
Brocherie et al. [8]	1	1	1	0	0	0	0	1	1	5
Cabrera Hernández et al. (2013)	1	1	1	0	0	0	0	1	1	5
Carling et al. [12]	1	1	1	1	0	1	1	1	1	8
Chmura et al. [14]	1	1	1	1	0	0	0	1	1	6
Chmura et al. [15]	1	1	1	1	0	1	1	1	1	8
Coker et al. [16]	1	1	1	1	1	1	0	1	1	8
Konefal et al. [22]	1	1	1	1	0	0	0	1	1	6
Konefal et al. [23]	1	1	1	1	0	0	1	1	1	7
Link and Weber [24]	1	1	1	1	1	0	0	1	1	7
McSharry [26]	1	1	1	0	0	0	0	1	1	5
Mohr et al. [27]	1	1	1	1	1	0	0	1	1	7
Nassis [28]	1	1	1	1	1	0	0	1	1	7
Nassis et al.[29]	1	1	1	0	0	0	0	1	1	5
Ozgünen et al. [30]	1	1	1	1	1	0	0	1	1	7
Tovar [34]	1	1	1	1	0	1	0	1	1	7
Trewin et al. (2018)	1	1	1	1	1	1	1	1	1	9
Watanabe et al. [36]	1	1	1	1	1	1	0	1	1	8
Zhou et al. [37]	1	1	1	0	0	0	1	1	1	6

## 3 Results

As described in the introduction due to the high complexity of the sport of soccer, many factors influence athletic performance and related competitive outcomes. While in research some authors include situational factors such as match location, match strength, result, position etc. as well as environmental factors [35,37]; e.g. Chmura et al. [15], other studies focus only on environmental factors (e.g. Chmura et al. [14]. In addition, different research objects were chosen, i.e. special leagues, competitions or teams. The number of players ranges from 6 to 1.644, with no specific number given in six publications. Along with this, the number of games also varies greatly from three to 2.039. The larger data sets refer to longer periods of time such as an entire tournament. The longest time span here is 104 years [26].

The most important environmental factors in the present publications are temperature, humidity and altitude. Of the 21 selected papers, six deal with the factor altitude [2,6,11,26,28,34], five with temperature [4,12,16,24,27], and one with both factors mentioned. Six papers examine humidity in addition to temperature [8,14,22,29,30,37], and only three studies include other factors such as cloud cover, wind or rain [15,23,36].

Referring to the dependent variables investigated, studies that focus on physical performance dominate. Mainly, the running performance (e.g. total distance covered, low/moderate/high intensity distance, number of sprints, top speed) of individual players or the whole team are analyzed [12]; e.g. Link and Weber [24]. In addition, some studies include the technical performance (e.g. shots, passes, tackles, possession, fouls, goals) of individual players or the whole team [37]; e.g. Chmura et al. [15]. In some cases, general variables relating to players and teams (e.g. age, market value, position, world ranking) are also included [8]; e.g. Watanabe et al. [36].

Table 5 summarizes all 21 analyzed studies, indicating the research object, the period of time, the technology or method used, the environmental factors considered (independent variable) and the dependent variables investigated. An overview on the results on physical and technical performance of each study is shown in Table 6. In the following, the most important changes in physical and technical performance are described more detailed.

**Table 5:** Overview of the selected studies.

Author (Year)	Research object	Period of time	Technology/Method	Environmental factor (Independent variable)	Physical performance (Dependent variable)	Technical performance (Dependent variable)
Aughey et al. [2]	20 Sea Level (Australian) and 19 Altitude-resident (Bolivian) professional male players	Five games, two near sea level (430 ​m) and three in La Paz (3600 ​m) in 2012	GPS (Catapult MinimaxX S4)	Altitude	Total distance, high speed run distance	–
Benjamin et al. [4]	19 female players of the NCAA Division I	26 matches in one season	GPS unit (Viper Pod, STATSports)	WBGT (temperature ​+ ​humidity)	Relative distance, relative high speed run distance, relative high metabolic load	–
Bohner et al. [6]	6 female players of the NCAA Division I	Three games, two at sea level (25 ​m) and one at a moderate altitude (1839 ​m) during three weeks in October	10-Hz GPS (Catapult, Minimax 4.3)	altitude	Total distance, high intensity running, % of time at HIR	–
Brocherie et al. [8]	6 male national teams in the Gulf Cooperation Council (GCC) region	2.008 matches from 1957 to 2012	Generalized linear model with a logit link function and multiple regression analysis	Temperature, humidity, WBGT (temperature ​+ ​humidity)	–	Results, goal difference
Cabrera Hernández et al. [11]	Male teams in the CONMEBOL Libertadores Cup	2.039 matches of the CONMEBOL Libertadores Cup from 2000 to 2015	Poisson's generalized linear model	Altitude	–	Results, number of goals
Carling et al. [12]	Nine male players of the 1st French League	166 matches from Season 2007/8 to 2010/11	Optical tracking (AMISCO Pro)	Temperature	total distance, distance in 3 intensities	–
Chmura et al. [14]	340 male players from 32 national teams	FIFA World Cup 2014	Castrol performance Index (Optical Tracking)	temperature, humidity	Total distance, distance in 3 intensities, number of sprints, top speed	–
Chmura et al. [15]	779 male players of the 1st German League	1.530 matches from Season 2014/15 to 2018/19	IMPIRE AG system	Temperature, humidity, precipitation	Total distance, top speed, sprint effort, high intensity effort	Shot, pass, duel, duel won/lost/succ, cross
Coker et al. [16]	7 male players of the NCAA Division I	12 matches from August to November	Heart rate monitors and GPS (Model BH3; Zephyr Technology Corporation)	Temperature	Total distance, distance in different intensities	–
Konefal et al. [22]	607 male players from 32 national teams	64 matches of the FIFA World Cup 2014	Castrol performance Index motion analysis system	Temperature, humidity	Total distance (match, first half, second half), distance in 3 intensities, number of sprints, top speed	–
Konefal et al. [23]	340 male players from 32 national teams	945 observations during FIFA World Cup 2018	STATS® motion analysis system	UTCI (air temperature, humidity, wind speed, cloudiness)	Total distance, distance with high intensity, number of sprints	Number of shots, number of passes, pass accuracy, final ranking places
Link and Weber [24]	Male players from 38 teams in the 1st and 2nd German League	1.211 matches from Season 2011/12 and 2012/13	Optical Tracking (VisTrack)	temperature	total distance	–
McSharry [26]	10 male national teams from South America	1.460 matches from 1900 to 2004	?	Altitude	–	Probability of a win, number of goals scored and conceded
Mohr et al. [27]	17 male players from two Scandinavian teams	Two experimental matches during six days	Optical tracking (AMISCO Pro)	Temperature	Total distance, distance in 2 intensities, 3 fatigue indexes, average and peak sprinting speed, sprint length	Successful passes/forward passes/crosses, number of passes/forward passes/crosses, average length, gain and loss of ball possession, challenges, sum of ground and air duels
Nassis [28]	Male players from 32 national teams	64 matches of the FIFA World Cup 2010	Optical tracking (not listed)	Altitude	Total distance, distance with/without ball possession, top speed	Goals scored per game, goals conceded due to goalkeepers’ error
Nassis et al. [29]	Male players from 32 national teams	64 matches of the FIFA World Cup 2014	Castrol Performance Index (Optical Tracking)	WBGT (temperature, humidity)	Total distance, distance in 4 intensities, top speed, number of sprints	Actual playing time, goals scored, yellow and red cards, passes (number and succes rate)
Ozgünen et al. [30]	11 male players from Ankara	Two competitive matches in June und July 2007	GPS (Forerunner 305)	Temperature, humidity	Total distance, distance in 4 intensities	–
Tovar [34]	722 male players from 32 national teams	126 games of the Copa Libertadores 2013	Opta	Altitude	–	Total passes, passes in opponents’ half, succesfull passes (number and percentage)
Trewin et al. [35]	45 female players from the same national team	47 competitive matches	GPS (Catapult MinimaxX S4)	Temperature, altitude	Total distance, distance in 2 intensities, number of sprints in 2 intensities	–
Watanabe et al. [36]	1.644 male players from 32 national teams	64 matches of the FIFA World Cup 2014	Matrics tracking system	Heat index (temperature ​+ ​humidity), wind, rain, cloud cover, altitude	Total distance, distance at high intensity, number of sprints	
Zhou et al. [37]	Male players from the Chinese Super League	240 matches from season 2015	Optical tracking (AMISCO Pro)	Temperature, humidity	Total distance, distance in 3 intensities, number of sprints in 3 intensities	Shot, ball possession, pass, position on the field, cross, corner, offside, 50–50 challenge won

**Table 6:** Main findings of the selected studies on physical and technical performance.

Author (Year)	Physical Performance	Technical Performance
Aughey et al.[2]	High Altitude (3.600) reduces the total distance and high-speed run distance covered during matches.	–
Benjamin et al. [4]	Statistically significant differences were observed in relative distance, relative high-speed run distance and relative high metabolic load for increased WBGT.	–
Bohner et al. [6]	The results indicate that teams residing at SL and competing at a moderate altitude may have a reduced ability in distance covered and a high intensity run rate.	–
Brocherie et al. [8]	–	In Gulf Cooperation Council (GCC) region, higher temperature increased the likelihood of a favorable outcome when playing against non-GCC teams.
Cabrera Hernández et al. [11]	–	The findings suggest that the away team is more likely to lose a match when it has to descend two or three altitude categories and when it ascends three altitude categories.
Carling et al. [12]	The present findings generally suggest that physical performance (total distance, distance run in 3 intensities) in professional soccer does not decrease in cold temperatures (<5 ​°C).	–
Chmura et al. [14]	Results presented indicate that the conditions most comfortable for physical activity on the part of players occur at 22 ​°C, and with relative humidity under 60%.	–
Chmura et al. [15]	From the range of environmental factors tested, only temperature affects physical activity, especially on total distance and number of sprints.	Only trivial effects were observed on technical performance in this league. Only Snowfall affects midfield positions in terms of the number of duels.
	Decreasing and/or increasing the humidity and WBGT beyond the comfortable range does not affect physical and technical activity. Similarly, the deterioration of ground wear and the deterioration of weather conditions do not show substantially negative effects. This indicates that professional players in the German Bundesliga do not modify and/or adopt their behaviour quickly with respect to different environmental conditions.	
Coker et al. [16]	The results indicate that heat stress conditions resulted in increased low-intensity running and heart rate, while high-intensity running was maintained. High-intensity running performance may be conserved through decreased playing time or the adoption of pacing strategies.	–
Konefal et al. [22]	Higher air temperature reduces the performance of exercise with medium and high intensity; while higher humidity has a significant positive impact on the distance covered by players with medium intensity. The total number of performed sprints becomes significantly lower in higher air temperature and humidity.	–
Konefal et al. [23]	Situations where the climatic conditions at the training centres indicate no thermal stress (UTCI between 9 and 26 ​°C) in comparison to real matches at the World Cup (with thermal stress) are more beneficial for increasing only the physical activity (total distance covered and number of sprints) of players.	The number of performed passes is better in conditions with no thermal stress in contrast to a higher UTCI with thermal stress. No significant effect was found for the number of shots and pass accuracy in relation to the UTCI category.
Link and Weber [24]	Data show a significant decrease in total distance covered from neutral (−4 to 13 ​°C) to warm (>14 ​°C) environments. The size of the temperature effect is greater in the 1st Bundesliga compared to the 2nd Bundesliga. No reduction in running performance due to cold (<5 ​°C) temperatures was observed.	–
McSharry [26]	–	High altitude teams score more and concede fewer goals with increasing altitude difference. Each additional 1000 ​m of altitude difference increases the goal difference by about half of a goal. The probability of the home team winning for two teams from the same altitude is 0.537, whereas this rises to 0.825 for a home team with an altitude difference of 3695 ​m and falls to 0.213 when the altitude difference is −3695 ​m.
Mohr et al. [27]	Total game distance and especially high intensity running were lower during a football game in the heat (43 ​°C), but these changes were not directly related to the absolute or relative changes in core or muscle temperature. However, peak sprinting speed was improved in the hot condition.	Execution of successful passes and crosses were improved in the hot condition (43°) in comparison to temperate conditions (21 ​°C).
Nassis [28]	Results show a 3.1% lower total distance that was covered by the teams during the matches played at 1200–1400 and 1401–1753 ​m compared with sea level. However, distances covered both with and without ball possession, and top running speed, did not differ between the game locations.	Indices of technical skills, including number of goals scored per game and errors made by the goalkeepers that resulted in goals conceded, did not differ with altitude.
Nassis et al.[29]	There was no difference in total distance covered between the matches played under different environmental stress categories. The number of sprints was lower in high than in moderate or low environmental stress but peak speed was unaffected. The distance covered at high intensity was also lower under high than low environmental stress.	There was no difference in actual playing time, number of goals scored and number of cards between the matches played under different environmental stress categories. Number of passes was not different but the rate of successful passes was higher under high than low environmental stress.
	Top-level players seem to modulate their activity pattern during matches in a hot and humid environment (i.e., less high-intensity but more low-intensity running and successful passes) to preserve the global match characteristics (i.e., similar actual playing time, total distance covered, peak running speed and goals scored).	
Ozgünen et al. [30]	In both games, there was a reduction in total distance from the first to the second half, but this was only 5% in the moderate heat game (34 ​°C, 38% humidity) and 15% in the high heat game (36 ​°C, 61% humidity).	–
Tovar [34]	–	The results show that the percentage of successful passes rises by about 5.6 percentage points, mostly driven by each player's behaviour in his own half compared for games played away above 2500 ​m (8202 feet) vis-à-vis those held below that threshold.
Trewin et al.[35]	At altitude (>500 ​m), a small increase in the number of accelerations and a small decrease in total distance were observed, whereas at higher temperatures, there were decreases in all metrics.	–
Watanabe et al. [36]	The results show that the heat index (combining temperature and humidity) significantly decreased running performance (number of sprints, high-intensity running), while a clear sky was positively associated with distance covered at high intensity. Wind speed has a significant negative effect on attacking. Rain was found to be insignificant in all models	–
Zhou et al. [37]	Increase in humidity would decrease the physical performance at a small magnitude. Teams achieved the most total distance, sprinting distance, sprinting effort, high-speed-running distance, high-speed-running effort, high-intensity-running distance and high-intensity-running effort at the temperature between 11.6 and 15.1 ​°C.	Increment in relative air humidity and air quality index would only bring trivial or small effects on all the technical performance. The teams had the highest number of shots, forward passes, offsides and fouls committed whilst playing at the temperature between 13 and 22 ​°C.
	Environmental factors affected mainly the physical performance but had only trivial or small impact on the technical performance.	

### 3.1 Physical performance

Physical performance in soccer can be evaluated using various parameters that can be measured over the entire game with the help of GPS tracking systems. Typical indicators of physical performance are the total distance covered, the distance covered at different intensities, the number of sprints or the maximum speed reached.

In all 17 studies that examined changes in physical performance due to environmental factors, the total distance covered per game was examined. Most of the studies observed a significant negative effect on total distance, both with increasing temperature (e.g. Refs. [24,27], and in combination with increasing humidity [30]; e.g. Benjamin et al. [4]. For example, Ozgünen et al. [30] compared a game at 34 ​°C and 38% humidity with a game at 36 ​°C and 61% humidity. They concluded that the first game averaged 4,386 ​m in the first half and 4,227 ​m in the second half, while the second game averaged only 4,301 ​m in the first half and 3,761 ​m in the second half. In contrast, other studies found no significant decrease in running performance with increasing temperature and humidity [22,29]. Other parameters, such as distance covered at high intensity or number of sprints, also showed no significant changes in some publications [16]; e.g. Chmura et al. [15] and decrease in some cases with more extreme environmental conditions [22]; e.g. Chmura et al. [14]. Only maximum velocity, which has been investigated by some studies, showed no significant changes under heat or increased humidity, with one negative exception [15] and one positive exception [27]. Low temperature (<5 ​°C) also did not significantly changed any of the physical parameters examined in [12].

Other factors such as cloud cover or rain were only investigated by [36] and showed no significant effects. Only an increased distance in high intensity with clear sky and a negative effect due to increased wind speed in attacking could be observed [36].

For the factor altitude, the results of the four studies examined agreed more clearly. Both total distance and distance covered at high intensity decreased significantly with increased altitude. In contrast, no significant changes were observed in the number of sprints and maximum speed under the influence of altitude. In Trewin et al. [35] even the number of sprints performed increased with a simultaneous decrease in total distance. Here, 47 matches of a women's national team were investigated, of which 40 were played near sea level (<500 ​m) and 7 ​at higher altitude (>500 ​m). For example, for the sea-level matches, the average total distance was 108 ​m/min, whereas for altitude this was only 104 ​m/min [35].

In summary, the studies identified by the systematic database search showed only partially significant changes in performance parameters due to changing environmental conditions. Especially the factor altitude seems to have a negative influence and also for heat in combination with increased humidity many studies showed a decreased physical performance.

According to [37] best physical performance was achieved at a temperature of 11.6–15.1 ​°C. Under these conditions, the human body can best adapt to the external conditions and can deliver the best physical and technical performance. Another study conducted by Chmura et al. [14] at the 2014 FIFA World Cup found optimal performance at a temperature below 22 ​°C and relative humidity below 60%. The worst conditions were found when the humidity remained above 60% at temperatures above 22 ​°C. However, when the temperature rises above 28 ​°C, humidity below 60% is more detrimental [14].

### 3.2 Technical performance

In addition to physical performance, the technical performance of a team and its individual players also has a significant influence on the outcome of a match. Typically, parameters such as goals, passes, shots, ball possession or duels are recorded using GPS tracking systems to evaluate technical performance. In the systematic database search, a total of 10 studies were identified that analysed the influence of environmental factors on technical performance.

In contrast to physical performance, it is striking that technical performance is only significantly changed by environmental factors in a few examples. For example, the number of shots was not affected by changing temperature [15] or humidity [37]. The same was seen in most studies that examined the number of successful passes. Here, however, rather significant positive effects could be observed in some cases. In a study of all FIFA World Cup 2014 matches the rate of successful passes was higher under high than low environmental stress [29]. Mohr et al. [27] also confirmed an improvement in successful passes and crosses in the hot condition (43°) in comparison to temperate conditions (21 ​°C). The authors therefore also suspected increased ball possession, but the results on gain and loss of ball possession were significantly negative for hot conditions [27]. A negative influence on the number of passes at a higher UTCI with heat stress was also found by Konefal et al. [23]. According to Zhou et al. [37] the best technical performance was achieved in the range of 13–22 ​°C.

Other environmental factors such as altitude [28] wind, cloud cover, or rain [15] showed only trivial effects on technical performance. Only snowfall has a significant negative effect on midfield positions in terms of the number of duels [15]. In contrast some positive changes were observed for the factor altitude. In a study of the Copa Libertadores 2013, the percentage of successful passes increased by 5.6% at altitudes above 2,500 ​m in contrast to sea-level matches [34]. In addition, high altitude teams score more goals and concede fewer goals with increasing altitude. Each additional 1000 ​m of altitude difference increases the goal difference by about half of a goal [26]. Thus, the authors come to different conclusions than [28]. An analysis of the 64 matches of the 2010 FIFA World Cup showed no significant changes for all indices of technical skills, including number of goals scored per game and errors made by the goalkeepers that resulted in goals conceded [28].

Another important point are possible advantages of teams that train and play in certain climatic conditions and face non-acclimatized teams. For instance [8], found that in Gulf Cooperation Council (GCC) region, higher temperature increased the likelihood of a favourable outcome when playing against non-GCC teams. This fact plays a role especially in World Cups, which can take place in countries with special environmental conditions, like Brazil 2014or Qatar 2022 [7].

## 4 Discussion

After describing all relevant results of the selected studies, the results will be summarized and discussed below based on the three most important environmental factors: temperature, humidity and altitude. Other factors were considered in only three studies. In general the studies, who analysed both physical and technical performance, find that all investigated environmental factors have a greater impact on the physical than the technical performance of players or the team [27,29,36,37]. Under hot conditions, the number of sprints was reduced in order to continue to maintain top speed as well as passing accuracy [14]. These results are consistent with the findings of Nassis et al. [29]; who observed no differences in technical performance like playing time, total distance, number of goals and cards, or number of passes as heat increased. On the other hand, the number of sprints and the distance covered with high intensity decreased, while the passing rate actually increased. Although physical performance was partially reduced, key performance indicators were maintained (top running speed) or improved (successful passes) as environmental heat stress increased [29]. Moreover, this conscious adaptation of the body to increased temperatures by reducing the total distance in order to continue to perform actions under high intensity at maximum speed was more evident in 1st Bundesliga matches compared to 2nd Bundesliga matches, although these players presumably have a higher level of fitness. This suggests that better players are more able to adapt their bodies to changing environmental conditions, ultimately increasing their overall performance [24]. Ozgünen et al. [30], who compared two games under different environmental conditions, adds that, if possible, players set their own pace to keep thermal stress within tolerable limits. An adoption of pacing strategies in the heat is also recommended by Coker [16]. In summary for both factors humidity and temperature it can be said, that top-level players seem to modulate their activity pattern during matches (less high-intensity but more low-intensity running and successful passes) to preserve the global match characteristics (similar actual playing time, total distance covered, peak running speed and goals scored) [29].

The results for the factor altitude were also similar. For example Tovar et al. [34] explain an improved pass rate with a simultaneous reduction of some physical parameters with the risk aversion of the players. They think about possible effects of altitude on their game and therefore take less risk when playing passes. Some authors attribute an advantage to teams adapted to height in home games, in relation to the end result [11] or the number of goals scored and conceded [26]. Thus, there does not seem to be an acclimatization advantage of players who play regularly on the continent [36]. In addition, the pre–World Cup training camps may have indeed been successful in adapting players to the climate, as shown in prior research [10].

## 5 Conclusion

To conclude this systematic review, this chapter formulates implications for sport practice, identifies limitations of the studies and this paper, and presents a summary.

### 5.1 Implications

The results of the analysed studies should be used in sports practice in order to be able to react to environmental influences in the best possible way. This applies on the one hand to the professionals and their functional team in training and competition [7,31,33] and on the other hand to the organizers of soccer events [23,24,36].

Regarding the factors heat and humidity, research offers numerous implications for training and competition environments. Soccer teams that are well adapted to elevated air temperature and humidity, and whose players are well hydrated, can maintain a high level of performance throughout the duration of the match and thus create more scoring opportunities [22]. In this regard, acclimatization with high-intensity exercises and sprints is recommended for increasing physical performance without causing motor and health problems for the players [14]. Since extreme environmental conditions affect players' speed rather than endurance skills, short-duration and explosive exercises should be included in training along with high-intensity exercises [22]. Players should also be sensitized to think about an intelligent movement strategy to achieve tactical goals with a minimum of physical effort [24]. They should not try to maintain their running performance at the same level as in colder conditions but adapt their pacing strategy by spending more time at lower movement speeds to maintain high intensity when needed [16].

Regarding the factor altitude, FIFA gives recommendations in its consensus statement on preparation with acclimatization and training concepts and on the prevention and treatment of acute altitude illnesses [3]. Another position statement provides a guide for support teams (coaches, performance scientists, physicians, strength and conditioning staff) and other professionals who have an interest in the practical application of altitude training for team sports [19]. Other publications also deals with altitude training in team sports [1,5,9], while other papers focus on acclimatization, with some different approaches [2,20,26,28]. Aughey et al. [2] for instance states that neither a thirteen-day acclimatization nor a lifetime stay at high altitude protects against the adverse effects of altitude on the activity profile of the game.

### 5.2 Limitations

Since some of the publications identified for this review differ greatly in their approaches, this also results in some limitations that can classify the works and provide pointers for further research.

Because only field studies of real competitions were considered, some authors criticize an insufficient or limited amount of data, for instance limited number of players [6,16], only a specific league [15], a specific time period [36] no positional differences [2,6,35]. Thus, expanding the data sets would be useful to check the robustness of the results and the influence of environmental factors on specific positions on the field.

Along with this, the size of the dataset also limits the investigation of other important influencing factors and their interactions [35,37]. For example, player performance may be influenced by the score, the system of play, the quality of training and coaching, or the relative strength of opposing teams [2,15,24,26]. Furthermore, external factors such as tactics, spectator support, location, water breaks, or travel effects may play an important role [8,16,29]. However, integrating too many independent variables may lead to an overly complex model with difficulties in interpretation [8].

Another point mentioned by some authors is the individual fitness level of the players [4,24]. Moreover, some authors lack information on additional environmental factors such as wind chill as well as changes in climatic conditions during the Games [11,12]. Furthermore, the recording of the environmental conditions can also be problematic. For example, in some cases no or limited data are available directly at the game location and data from the nearest local weather station must be used [4].

In addition to the field studies examined in this review, laboratory studies should also be included, especially to better control performance diagnostics [2,6,12]. These also provide a good basis for exploring other topics, such as the effects of environmental factors on player health (e.g., dehydration, hyperthermia, frequency of injury) [36] or adaptation strategies to specific environmental conditions [12].

### 5.3 Summary

The systematic literature search on the effects of selected environmental factors on physical and technical performance in professional soccer identified 21 relevant studies from 2.396 records in the databases. These were mainly concerned with the factors of temperature, humidity and altitude, and their effects on physical performance dominate over technical performance. The studies differed greatly with respect to research object, number of players and games studied and the associated time duration. For all analysed environmental factors it can be summarized that in different conditions, professional soccer players may consciously adjust certain performance parameters to maintain key match characteristics throughout the whole game. This pacing strategy allows them to keep the influence of environmental factors in check as far as possible.

Even though most weather factors cannot be influenced by humans, knowledge about these effects in sports helps to better assess the influence of environment on the sports market. This knowledge can be used by decision-makers in sports to minimize risks associated with weather and to use potential opportunities to their advantage. At this point in time, there is already some work that deals primarily with hot and cold conditions as well as humidity and altitude in sports. Other weather elements such as precipitation or wind, however, have not yet been given much attention and therefore offer potential for further research. Interactions between individual environmental factors as well as the influence of other external factors on performance in soccer have also been insufficiently studied.

## Declaration of interest statement

The authors declare that the research was conducted in the absence of any commercial or financial relationships that could be construed as a potential conflict of interest.
